# Oxidative Stress Markers Are Lower in MINOCA Than in MI-CAD, Despite Comparable Inflammatory Status

**DOI:** 10.3390/antiox14040449

**Published:** 2025-04-09

**Authors:** Haldun Koç, Ahmet Seyda Yılmaz, Karolin Yanar, Abuzer Duran, Müjgan Ayşenur Şahin, Muhammed Mürsel Öğütveren, Yusuf Hopaç

**Affiliations:** 1 Department of Cardiology, Faculty of Medicine, Recep Tayyip Erdogan University, Rize 53100, Türkiye; haldunkoc74@gmail.com (H.K.); abuzer.duran6@gmail.com (A.D.); aysenurcin@hotmail.com (M.A.Ş.); muhammedr4648@gmail.com (M.M.Ö.); yusuf809@gmail.com (Y.H.); 2 Department of Medical Biochemistry, Faculty of Medicine, Istanbul University-Cerrahpaşa, Istanbul 34098, Türkiye; karolin.yanar@iuc.edu.tr

**Keywords:** MINOCA, oxidative stress, inflammation, coronary artery disease

## Abstract

Myocardial infarction (MI) is defined as a clinical event in which myocardial damage is evidenced in the setting of myocardial ischemia. However, patients without occlusive coronary artery stenosis can also have myocardial infarction, which is titled Myocardial Infarction with Non-Obstructive Coronary Arteries (MINOCA). In our study, we aimed to evaluate oxidative stress and inflammation responses between MINOCA and MI with coronary artery disease (CAD) patients. In this prospective, cross-sectional study, patients with elevated cardiac markers who were admitted to the cardiology clinic between March 2024 and May 2024 with the preliminary diagnosis of acute coronary syndrome were included. Patients were consecutively collected as those with an occlusive lesion on coronary angiography and those without. Routine blood samples and oxidative stress parameters were obtained and compared between groups. A total of 88 patients, including 44 MINOCA and 44 MI-CAD patients, were included in the study. The MINOCA group was significantly younger than the MI-CAD group (56.2 ± 12.5, vs. 64.7 ± 9.3, *p*: 0.001). While inflammatory parameters were similar between groups, dityrosine (5708 FU/mL (5311–6417) vs. 4488 FU/mL (3641–5238), *p* < 0.001), lipid hydroperoxide (3.6 nmol/mL (3.4–3.9) vs. 3.4 nmol/mL (3.1–3.9), *p*: 0.023), kynurenine (3814 ± 621 FU/mL vs. 3319 ± 680 FU/mL, *p*: 0.001), and malondialdehyde (17.4 nmol/mL (13.7–19.1) vs. 13.1 nmol/mL (12–14.9), *p* < 0.001) levels were higher in the MI-CAD group than in the MINOCA group. Although inflammation parameters did not differ between MI-CAD and MINOCA patients, oxidative stress parameters were higher in the MI-CAD group. Regardless of the presence and severity of inflammation, oxidative markers can help to assess the level of myocardial cell damage, risk stratification, and diagnosis of myocardial infarction.

## 1. Introduction

Myocardial infarction (MI) is responsible for approximately 18 million deaths annually worldwide and continues to cause significant short- and long-term morbidity due to complications such as heart failure, valvular heart diseases, arrhythmias, cerebrovascular events, organ failures, and many others. Consequently, ongoing research focuses not only on the incidence and prevalence of MI but also on identifying its underlying causes, understanding its pathophysiological stages, and developing preventive strategies [1].

While about 90% of myocardial infarctions are attributed to obstructive coronary artery disease (CAD) caused by atherosclerotic plaques, MI can also occur in the absence of obstructive coronary artery stenosis. This clinical phenomenon, increasingly observed in recent years, was introduced to the literature by Beltrame et al. under the term “Myocardial Infarction with Non-Obstructive Coronary Artery” (MINOCA) [2]. MINOCA encompasses a heterogeneous group of patients with varied etiologies, pathophysiological mechanisms, and treatment approaches. Despite having a lower mortality rate compared to MI with obstructive CAD (MI-CAD), MINOCA is associated with considerable cardiovascular risks, including recurrent events, endothelial dysfunction, and other adverse outcomes [3]. This necessitates ongoing studies to better understand its underlying causes, diagnostic methods, and treatment options.

Inflammation plays a pivotal role in the pathophysiology of MI, making it a critical focus in diagnostic and therapeutic approaches. Inflammatory biomarkers such as the systemic immune-inflammation index (SII), C-reactive protein-to-albumin ratio (CAR), and neutrophil-to-lymphocyte ratio (NLR) have acquired attention for their ability to efficiently assess systemic inflammatory responses [4]. These indices provide valuable prognostic and diagnostic insights, facilitating risk stratification and guiding treatment strategies for both cardiovascular and non-cardiovascular events, including those in MI patients. Additionally, oxidative stress markers such as dityrosine, lipid hydroperoxide, kynurenine, and malondialdehyde have been linked to endothelial dysfunction, inflammation, and the severity of myocardial damage [5,6,7,8]. However, in patients with MI-CAD and MINOCA, comprehensive evaluations addressing oxidative stress remain inadequate.

In this study, we aim to compare the demographic characteristics, physical examination findings, laboratory results, and clinical data of MINOCA and MI-CAD patient groups. Furthermore, we aim to achieve a comprehensive understanding of the interplay between oxidative stress and inflammation in these clinically overlapping yet pathophysiological distinct entities. We also aim to provide deeper insights into the underlying mechanisms differentiating these patient groups to enlighten more precise diagnostic, prognostic, and therapeutic strategies.

## 2. Materials and Methods

### 2.1. Study Design

This prospective, cross-sectional study included patients over 18 years of age who were admitted to the cardiology clinic with a preliminary diagnosis of acute coronary syndrome, presented with elevated cardiac markers, and underwent coronary angiography between March 2024 and May 2024. The study was conducted following approval by the Non-Interventional Clinical Research Ethics Committee under protocol number 2024/72. All participants were informed about the study, and written informed consent was obtained from each patient.

### 2.2. Exclusion Criteria

Exclusion criteria included patients with complications such as PCI-related coronary dissection, coronary rupture, acute or hyperacute stent thrombosis, previous cardiovascular surgery for any indication, end-stage liver or kidney disease, malignancy, history of radiotherapy or chemotherapy, collagen vascular diseases, history of cerebrovascular events, endocrine disorders, active or chronic inflammatory diseases, moderate-to-severe valvular heart diseases, Takotsubo cardiomyopathy, acute or chronic pulmonary embolism, central nervous system pathologies, those with normal cardiac biomarkers, individuals under 18 years of age, and patients who refused to undergo angiography.

### 2.3. Physical Examination and Clinical Data Collection

Comprehensive physical examinations were conducted including demographic data such as age, sex, body mass index (BMI), smoking status, and comorbidities, including diabetes mellitus, hypertension, and dyslipidemia, were recorded. Medical history, prior use of medications, and prior cardiovascular events were also documented for both patient groups. Xanthelasma was identified as the most common cutaneous presentation of xanthomas caused by cholesterol accumulation in the skin, predominantly around the eyelids. These lesions were observed as semi-solid, yellowish papules or plaques and were characterized by deposits rich in soft lipids, primarily cholesterol. In addition, the degree of hair whitening was graded using a standardized scoring system applied only to male patients. The progression of whitening was categorized into five grades based on the proportion of black to white hair: Grade 1 (completely black), Grade 2 (predominantly black with some white), Grade 3 (equal proportions of black and white), Grade 4 (predominantly white with some black), and Grade 5 (completely white). Alopecia was also assessed only in male participants and classified according to the Norwood–Hamilton classification system during physical examination to determine the extent of hair loss. Although the Norwood classification includes seven grades, in this study, individuals with grade 5 and higher were collectively categorized as grade 5. Moreover, the presence of an ear lobe crease, also known as Frank’s sign, defined as a diagonal line running from the tragus to the earlobe at a 45° angle, was examined. Additionally, blood samples were collected in fasting conditions for routine biochemical and specialized oxidative stress marker analyses.

### 2.4. MI-CAD Definition

STEMI diagnosis was made based on the following criteria: the presence of ST-segment elevation greater than 1 mm in two contiguous leads on the ECG. (The threshold value for this criterion differs in chest leads V2 and V3 based on gender and age; it is ≥2 mm in men over 40 years, ≥2.5 mm in men under 40 years, and ≥1.5 mm in women regardless of age.) It should also be noted that posterior MI may present differently on ECG; in patients suspected of myocardial infarction with ST-segment depression in leads V1–V3, the diagnosis is established by observing ≥ 0.5 mm ST-segment elevation in leads V7–V9 obtained using electrodes placed on the back.

NSTEMI diagnosis was made based on the following criteria: patients presenting with MI symptoms without ST-segment elevation on the ECG at admission but with elevated cardiac biomarkers were classified as NSTEMI. There may be mild abnormalities on the ECG, such as ST-segment depressions and T-wave changes, but the ECG can also appear entirely normal.

### 2.5. MINOCA Diagnoses

The diagnosis of Myocardial Infarction with Non-Obstructive Coronary Arteries (MINOCA) was established based on the rigorous application of diagnostic criteria outlined in the Fourth Universal Definition of Myocardial Infarction. Acute myocardial infarction was identified through the detection of a significant rise and/or fall in cardiac biomarkers, with at least one measurement exceeding the 99th percentile of the upper reference limit. This biomarker evidence was accompanied by clinical and diagnostic findings indicative of myocardial injury, including one or more of the following: symptoms consistent with myocardial ischemia, new ischemic changes observed on the electrocardiogram (ECG), the development of pathological Q waves on the ECG, imaging evidence of new loss of viable myocardium, or new regional wall motion abnormalities. A critical component of MINOCA diagnosis was the exclusion of obstructive coronary artery disease on angiography among patients admitted with a preliminary diagnosis of ACS (acute coronary syndrome). Non-obstructive coronary arteries were defined as the absence of significant coronary stenosis, specifically less than 50% luminal narrowing, in any artery that could be implicated in the infarction.

### 2.6. Coronary Angiography and Percutaneous Coronary Intervention

Coronary angiography was conducted urgently using the Judkins technique in all patients. Left anterior descending (LAD) and circumflex (Cx) coronary arteries were viewed from at least four different angles and the right coronary artery (RCA) from at least two different angles. The coronary artery with total occlusion was revascularized with the coronary balloon and/or stent immediately after imaging. Thrombolysis in Myocardial Infarction (TIMI) flow was evaluated. In epicardial arteries with a diameter of ≥1.5 mm, any lesion that caused at least 50% lumen narrowing compared to the closest segment was considered a significant stenosis. Intervention to the total occluded coronary artery at first angiogram was determined as the revascularization strategy. In the case of a severe lesion other than the culprit lesion, elective PCI was planned. Patients were treated following the current guidelines and waited in the coronary intensive care unit until stabilization was achieved.

### 2.7. Laboratory Analysis

Suitable samples for routine biochemistry, hemogram, creatinine kinase-MB (CK-MB), troponin, C-reactive protein (crp), and albumin were obtained from all patients at admission. CK-MB and troponin-I (Tn-I) measurements were repeated at 4 h intervals. Additional blood samples obtained at admission were immediately centrifuged, and the obtained serum specimen was stored in a freezer at −40 °C during the study period. Oxidative stress parameters were analyzed through these materials.

### 2.8. Inflammatory Biomarkers

Crucial systemic inflammatory and nutritional markers, including the systemic immune-inflammation index (SII), C-reactive protein-to-albumin ratio (CAR), neutrophil-to-lymphocyte ratio (NLR), albumin-to-creatinine ratio (ACR), prognostic nutritional index (PNI), and platelet-to-lymphocyte ratio (PLR), were calculated from complete blood count and routine biochemistry results. The systemic immune-inflammation index (SII) was determined using the formula: (neutrophils × platelets)/lymphocytes. The PIV, reflecting overall immune and inflammatory activity, was calculated using the formula: (neutrophils × monocytes × platelets)/lymphocytes. Additionally, the prognostic nutritional index (PNI), which assesses the nutritional and inflammatory status of patients, was calculated using the formula: 10 × serum albumin (g/dL) + 0.005 × total lymphocyte count (per mm^3^). These indices collectively provided a comprehensive evaluation of systemic inflammation, immune response, and nutritional status, serving as valuable tools for risk stratification and prognostic assessments in the study population.

### 2.9. Evaluation of Oxidative Stress Parameters

Dityrosine is a marker of protein oxidation resulting from cross-linking of tyrosine residues and was analyzed using the spectrofluorometric method. Fluorescence measurements of the samples were recorded at excitation and emission wavelengths of 330 nm and 415 nm, respectively [9].

Kynurenine is a metabolite of the tryptophan degradation pathway, linked to oxidative and immune responses and analyzed with the spectrofluorometric method; fluorescence measurements of the samples were recorded at excitation and emission wavelengths of 365 nm and 480 nm, respectively [9].

Malondialdehyde (MDA) is a product of lipid peroxidation and a widely used biomarker of oxidative stress. The analysis was conducted using the method in which proteins in the samples were precipitated with trichloroacetic acid, followed by heating the samples with thiobarbituric acid and hydrochloric acid at 95 °C. The absorbance of the resultant pink complex was measured at a wavelength of 532 nm, and concentrations were calculated using the molar extinction coefficient [9].

Lipid hydroperoxide is an unstable primary product of lipid oxidation and indicates early oxidative damage to lipids. The analysis was conducted spectrophotometrically by adding 950 µL of FOX 2 reagent to 50 µL of the sample in tubes, followed by mixing using a vortex mixer. After 30 min of incubation, the samples were centrifuged, and the absorbance was measured at a wavelength of 560 nm. The concentration was calculated using the extinction coefficient [10].

### 2.10. Transthoracic Echocardiography

Detailed two-dimensional echocardiography was performed on all patients prior to discharge and during routine follow-up using a Philips Epiq 7 system (Andover, MA, USA) equipped with a 2.5–3.5 MHz transducer. Measurements of left atrial and ventricular dimensions, as well as septal and posterior wall thickness, were obtained via M-mode echocardiography. Additionally, conventional Doppler and Tissue Doppler parameters were recorded. Left ventricular ejection fraction (LVEF) was calculated based on left ventricular dimensions. Aortic valve calcification and thickening were defined as aortic valve sclerosis, while thickening and increased opacity over the posterior mitral valve leaflet were identified as mitral annular calcification (MAC).

### 2.11. Statistical Analysis

Statistical analyses were conducted using SPSS software version 22.0 (SPSS Inc., Chicago, IL, USA). The normality of continuous variables was assessed using the Kolmogorov–Smirnov test and visual inspections of Q-Q plots. Data were expressed as the mean ± standard deviation for normally distributed continuous variables, and as the median with interquartile ranges for skewed continuous variables. Categorical variables were presented as frequencies and percentages. Comparisons between the two groups were performed using chi-square tests for categorical variables, and Mann–Whitney U or independent samples *t*-tests for continuous variables, as appropriate. A *p*-value of less than 0.05 was considered statistically significant for all analyses. The relationship between MINOCA/MI-CAD and all coronary risk factors was analyzed. Factors showing significant differences between patient groups were subsequently included in univariate logistic regression analysis. Variables with an unadjusted *p*-value of <0.05 in the univariate logistic regression were identified as potential risk markers and were incorporated into a multivariate logistic regression model. Logistic regression analysis was thus employed to predict the diagnosis of MINOCA and MI-CAD. Additionally, Spearman’s rank correlation coefficient was used to evaluate correlations between inflammatory parameters and oxidative stress markers. A two-tailed *p*-value of <0.05 was considered statistically significant.

## 3. Results

### 3.1. Baseline Demographic and Clinical Characteristics

A total of 88 patients meeting the inclusion criteria were enrolled in the study, comprising 44 MINOCA patients and 44 MI-CAD patients. The mean age of all participants was 60.4 ± 11.7 years. Patients in the MINOCA group were significantly younger compared to those in the MI-CAD group (56.2 ± 12.5 vs. 64.7 ± 9.3, *p* = 0.001). Among all participants, 60 (68.2%) were male, with the proportion of males being higher in the MI-CAD group compared to the MINOCA group (34 (77.3%) vs. 26 (59.1%), *p* = 0.054). The overall body mass index (BMI) was 29.5 ± 4.5, and while the BMI of the MINOCA group was higher than that of the MI-CAD group, the difference did not reach statistical significance (30.5 ± 4.1 vs. 28.6 ± 4.7, *p* = 0.052). Waist circumference was 103.5 ± 13.1 cm across all patients, with no significant difference observed between the two groups (104.8 ± 11.6 vs. 102.1 ± 14.4, *p* = 0.322).

Hypertension was present in 54% (n = 48) of all patients, with no significant difference between the groups (25 (56.8%) vs. 23 (52.3%), *p* = 0.415). The overall prevalence of diabetes mellitus was 28% (n = 25), with no significant difference between the two groups (15 (34.1%) vs. 10 (22.7%), *p* = 0.172). The smoking rate among all patients was 42%, which was significantly higher in the MI-CAD group than in the MINOCA group (24 (54.5%) vs. 13 (29.3%), *p* = 0.015). Dyslipidemia was present in 17% (n = 15) of the overall patients, with a higher prevalence in the MI-CAD group compared to the MINOCA group (8 (18.2%) vs. 7 (15.9%), *p* = 0.05). A history of CAD was documented in 21 patients (23.9%), which was significantly higher in the MI-CAD group compared to the MINOCA group (16 (36.4%) vs. 5 (11.4%), *p* = 0.006) (Table 1).

### 3.2. Physical Examination Findings

Xanthelasma was observed completely in the MI-CAD group and was not present in the MINOCA group. There was no significant difference between the two groups in the prevalence of ear lobe crease (70.5% (n = 31) vs. 65.9% (n = 29), *p* = 0.410). Similarly, no significant difference was observed between the groups in the prevalence of anterior tragal crease (72% (n = 32) vs. 65% (n = 29), *p* = 0.322) (Table 1).

**Figure 1:**
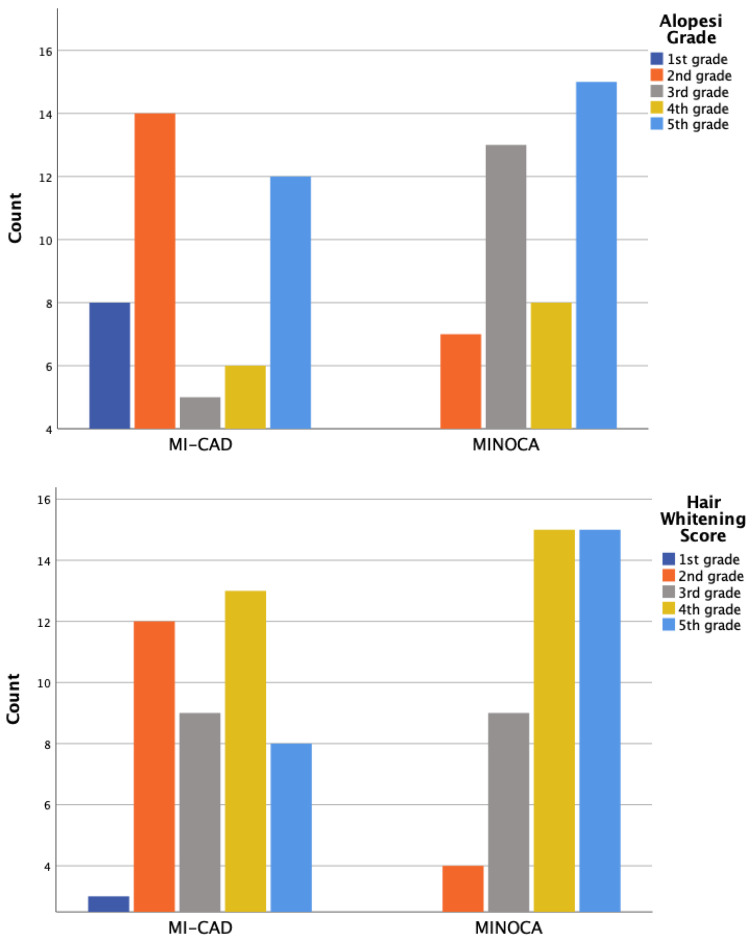
The distribution of hair whitening degrees and hair loss degrees (alopecia grade) based on the number of patients.

Alopecia and hair whitening assessments were conducted only in male patients, using a 5-point visual grading scale ranging from 0 (no visible change) to 5 (extensive involvement). Scoring was based on the extent and distribution of hair loss and whitening observed across the scalp. Moderate-to-severe hair whitening, defined as grade 3 and above, was observed in 81.7% of male participants. The prevalence was higher in the MI-CAD group compared to the MINOCA group (87.9% vs. 74.1%), although the difference was not statistically significant (*p* = 0.197). Moderate-to-severe alopecia, defined as grade 4 and above, was present in 88.3% of male patients. The MI-CAD group showed a significantly higher frequency compared to the MINOCA group (97% vs. 77.8%, *p* = 0.039) (Figure 1).

### 3.3. Echocardiographic Findings

The ejection fraction (EF) was significantly higher in the MINOCA group compared to the MI-CAD group (60% (60–60) vs. 53% (43–60), *p* < 0.001). The epicardial adipose tissue thickness was significantly greater in the MI-CAD group (0.5 mm (0.4–0.6) vs. 0.3 mm (0.3–0.4), *p* < 0.001). Aortic valve sclerosis was statistically more prevalent in the MI-CAD group compared to the MINOCA group (14 (31%) vs. 6 (13.6%), *p* = 0.037). Although mitral annular calcification (MAC) was more frequently observed in the MINOCA group, the difference was not statistically significant (*p* = 0.5).

Tissue Doppler findings revealed that the left ventricular lateral a’ wave (0.11 (0.09–0.13) vs. 0.10 (0.08–0.12), *p* = 0.024), lateral e’ wave (0.1 (0.08–0.13) vs. 0.08 (0.06–0.1), *p* = 0.012), tricuspid annular plane systolic excursion (TAPSE) (19 (17–21) vs. 20 (19–21.7), *p* = 0.043), right ventricular diameter (30 (27.5–33) vs. 28 (24–30), *p* = 0.04), right atrial diameter (33.5 (32–37) vs. 33 (30–34), *p* = 0.026), and right atrial maximal volume (34.5 (26–44) vs. 27 (20–33), *p* = 0.001) were significantly larger in the MINOCA group compared to the MI-CAD group.

The maximum and minimum left atrial volumes were higher in the MINOCA group compared to the MI-CAD group. Although this difference did not reach statistical significance, the *p*-value (0.054) indicates a trend toward significance, suggesting that the higher maximum left atrial volume observed in the MINOCA group may have potential clinical relevance. The minimum left atrial volume was higher in the MINOCA group compared to the MI-CAD group, but the difference was not statistically significant (*p* = 0.748). Similarly, the left atrial volume index (LAVI) was larger in the MINOCA group, yet this parameter did not reach statistical significance (*p* = 0.154) (Table 2).

### 3.4. Laboratory Findings

The total protein level was significantly higher in the MINOCA group compared to the MI-CAD group (73 mg/dL (70–76) vs. 69 mg/dL (66–73), *p* < 0.001). Otherwise, no significant differences were observed between the two groups for other blood parameters (Table 3).

### 3.5. Immunity-Inflammation and Oxidative Stress Parameters

There were no statistically significant differences between the MI-CAD and MINOCA groups in terms of SII, CAR, and NLR values. Although the MI-CAD group tended to have slightly higher median values across all three parameters, none of these differences reached statistical significance (*p* > 0.05 for all). These findings suggest that systemic inflammation, as captured by these indices, may not be the key differentiating factor between MI-CAD and MINOCA in this cohort. On the other hand, the levels of dityrosine (5708 FU/mL (5311–6417) vs. 4488 FU/mL (3641–5238), *p* < 0.001), lipid hydroperoxide (3.6 nmol/mL (3.4–3.9) vs. 3.4 nmol/mL (3.1–3.9), *p* = 0.023), malondialdehyde (17.4 nmol/mL (13.7–19.1) vs. 13.1 nmol/mL (12–14.9), *p* < 0.001), and kynurenine (3954 FU/mL (3317–4181) vs. 3206 FU/mL (2853–3765), *p* = 0.001) were significantly higher in the MI-CAD group (Table 4).

### 3.6. Independent Predictors of Myocardial Infarction with Coronary Artery Disease

Multivariable regression analysis identified age (OR: 1.195, 95% CI: 1.042–1.371, *p* = 0.011), smoking (OR: 76.919, 95% CI: 4.665–1263.774, *p* = 0.002), dityrosine (OR: 1.001, 95% CI: 1.000–1.002, *p* = 0.011), malondialdehyde (OR: 2.096, 95% CI: 1.286–3.416, *p* = 0.003), and increased right atrial maximum volume (OR: 0.740, 95% CI: 0.607–0.902, *p* = 0.003) as independent predictors of MI-CAD in patients admitted with a preliminary diagnosis of ACS (Table 5).

### 3.7. Independent Predictors of MINOCA

In multivariable regression analysis, smoking (OR: 0.009, 95% CI: 0.001–0.276, *p* = 0.007), right atrial maximum volume (OR: 1.365, 95% CI: 1.093–1.705, *p* = 0.006), dityrosine (OR: 0.999, 95% CI: 0.998–1.000, *p* = 0.026), and malondialdehyde (OR: 0.607, 95% CI: 0.382–0.963, *p* = 0.034) were identified as independent predictors of MINOCA (Table 6).

### 3.8. Correlation Analysis Between Inflammatory and Oxidative Stress Parameters

Correlation analysis was performed between inflammatory and oxidative stress markers. A positive correlation was observed between dityrosine and the systemic immune-inflammation index (SII) (r = 0.245, *p* = 0.021) as well as between dityrosine and the neutrophil-to-lymphocyte ratio (NLR) (r = 0.256, *p* = 0.016). Similarly, kynurenine showed a positive correlation with SII (r = 0.242, *p* = 0.023) and NLR (r = 0.276, *p* = 0.009).

### 3.9. Correlation Between Maximum Troponin, Ejection Fraction, and Inflammatory and Oxidative Stress Parameters

When analyzing the correlation between maximum troponin and oxidative stress parameters, significant positive correlations were observed with dityrosine (r = 0.389, *p* < 0.001), malondialdehyde (r = 0.228, *p* = 0.011), and kynurenine (r = 0.289, *p* = 0.007). In contrast, ejection fraction (EF) showed significant negative correlations with dityrosine (r = −0.220, *p* = 0.04), malondialdehyde (r = −0.339, *p* = 0.001), and kynurenine (r = −0.364, *p* < 0.001).

## 4. Discussion

We demonstrated in the current study that oxidative stress markers were elevated in MI-CAD patients despite similar inflammatory parameters being found between the two groups. We postulate that oxidative stress markers may predict cellular involvement with higher sensitivity than the well-established inflammatory biomarkers known to play a crucial role in the atherosclerotic process. Furthermore, although oxidative stress levels were lower in MINOCA patients compared to MI-CAD patients, the presence of cellular damage was also evident in the MINOCA group.

Myocardial infarction is defined as a clinical or pathological event characterized by myocardial damage within the context of myocardial ischemia. Diagnosis is established when typical symptoms, suspicious electrocardiographic findings, new loss of viable myocardium, or new regional wall motion abnormalities are accompanied by a rise and/or fall in troponin levels [11]. Myocardial infarction with non-obstructive coronary arteries (MINOCA) is defined as acute myocardial infarction in the absence of significant coronary artery stenosis (≤50%) on coronary angiography. In some cases, patients have been mistakenly diagnosed with ST-segment elevation myocardial infarction due to the absence of significant coronary artery thrombosis or stenosis on angiography. This compressed clinical scenario led to the initial definitions of MINOCA, highlighting the need for further research to elucidate its etiology, diagnostic protocols, and therapeutic approaches [12,13]. To date, several potential etiologies have been proposed for MINOCA, including coronary artery spasm, coronary microvascular dysfunction, plaque disruption, spontaneous coronary thrombosis or embolism, spontaneous coronary artery dissection, or cardiomyopathies [14]. Therefore, the fundamental principle in managing this syndrome lies in elucidating the underlying mechanisms to provide tailored treatments for each patient.

Atherosclerosis is the most common form of large vessel pathology responsible for ischemic damage syndromes in vital organs, such as myocardial infarction. The fundamental pathophysiological process of atherosclerosis involves chronic inflammation, where oxidative stress plays a significant role in regulating vascular homeostasis, including endothelial and smooth muscle cell growth, proliferation, and migration [15]. Within this context, biomarkers are frequently used to determine sensitivity and potential effects at both individual and population levels, to identify cause–effect and dose–response relationships, to assess exposures, and for diagnosis, treatment, and follow-up [16,17]. Numerous studies in recent years have highlighted the critical role of oxidative stress in the development of atherosclerosis and ischemia-reperfusion injury [18,19]. In our study, oxidative stress markers, dityrosine, lipid hydroperoxide, malondialdehyde, and kynurenine were found to be elevated in the MI-CAD group despite a similar inflammatory response between MI-CAD and MINOCA patients. These findings suggest that, while inflammation findings were comparable between groups, the toxic effects of oxidative stress were more noticeable at the cellular level in MI-CAD patients.

Oxidative stress contributes to endothelial dysfunction, atherosclerosis, dyslipidemia, hypertension, diabetes, chronic kidney disease, heart failure, and ischemia/reperfusion injury, playing a critical role in the pathogenesis of numerous cardiovascular diseases [20]. Furthermore, oxidative stress is pivotal during the restoration period following ischemia-reperfusion injury. In addition to causing endothelial cell damage, it provides more detailed information about microvascular pathologies, such as vascular tone and reactivity, tissue hypoxia, and fibrosis, compared to inflammatory markers. Today, oxidative stress is recognized as a novel risk factor responsible for the development of CAD, affecting both prognosis and quality of life [21,22]. Our study also demonstrated that oxidative stress parameters correlated more strongly with reduced EF than inflammatory parameters across all patient groups. Notably, one of the oxidative stress markers, dityrosine, was significantly elevated in the MI-CAD group. Dityrosine concentrations in LDL isolated from human atherosclerotic lesions were found to be 100 times higher than those in LDL isolated from healthy subjects [23]. Mayer et al. showed an inverse correlation between dityrosine levels and survival rates in MI patients, and they concluded that dityrosine could be detected post-infarction and might aid in early diagnosis where time is critical [24]. Thus, it has been proposed that dityrosine levels may predict cardiac event risk. Another oxidative stress parameter, malondialdehyde (MDA), was also found to be significantly elevated in the MI-CAD group. Recent findings demonstrated a relationship between MDA and common cardiovascular risk factors. Hypertension, lipid imbalances, and diabetes were associated with increased MDA concentrations [25]. Furthermore, recent studies have shown that kynurenine and its metabolites play a prominent role in the pathophysiology of cardiovascular diseases and that pharmacological interventions targeting the kynurenine pathway may have therapeutic value in various cardiovascular diseases. Overactivation of the kynurenine pathway has been linked to a higher risk of acute myocardial infarction, particularly in diabetic and prediabetic patients with stable angina pectoris [26]. In addition, Dschietzig et al. found higher plasma kynurenine levels in patients with congestive heart failure (CHF), particularly those with higher New York Heart Association (NYHA) functional class scores [27]. Comparably, in our study, kynurenine levels were significantly elevated in the MI-CAD group. These findings may partly explain why the MI-CAD patient group has a higher probability of adverse events compared to the MINOCA patients.

Lipid peroxidation plays a pivotal role in the development of atherosclerosis, where reactive oxygen species (ROS) oxidize unsaturated lipids, producing a diverse range of oxidation products. These molecules can interact with circulating lipoproteins, penetrate cells, and even cross biological membranes. Furthermore, they can modify nucleophilic regions within biomolecules such as DNA, lipids, and proteins, leading to numerous biological effects. Lipoxidation products are no longer viewed merely as markers of oxidative damage but are also considered potential therapeutic targets due to their involvement in the pathogenic mechanisms of oxidative-related diseases, such as diabetes, atherosclerosis, and neurological disorders. Que et al. proposed that therapies targeting oxidized lipids could be beneficial in mitigating generalized inflammation, including the progression of atherosclerosis, aortic stenosis, and hepatic steatosis [28]. In line with these findings, our study observed significantly higher lipid hydroperoxide levels in the MI-CAD group, reflecting vaster cellular damage at the molecular level.

On the other hand, significantly elevated levels of oxidative stress markers in MI-CAD patients highlight these molecules as potential therapeutic targets. Recent studies have identified several therapeutic compounds capable of modulating oxidative stress markers. Notably, inhibitors of soluble epoxide hydrolase have emerged as promising agents in regulating redox homeostasis. These inhibitors reduce dityrosine accumulation by limiting oxidative modifications of protein tyrosine residues, which are mediated by reactive nitrogen species such as peroxynitrite [29]. In addition, several compounds target the kynurenine pathway to reduce oxidative stress in cardiovascular disease. Among them, Indoleamine 2,3-dioxygenase inhibitors, kynurenic acid analogs, and melatonin have shown therapeutic potential in modulating kynurenine metabolism and reducing myocardial damage [30]. Moreover, statins and omega-3 fatty acids have been reported to decrease serum malondialdehyde concentrations, potentially alleviating oxidative stress-related myocardial injury [31]. Similarly, lipid hydroperoxides are attenuated by compounds like carvedilol and statins, which interrupt the lipid peroxidation cascade and reduce oxidative burden in cardiovascular tissues [32]. The identification of these compounds provides a potential foundation for personalized antioxidant strategies and therapeutic decision making based on oxidative biomarker profiling in MI-CAD patients.

While revascularization remains the cornerstone therapy for MI-CAD, it is not a therapeutic option for MINOCA; selected patients may require urgent interventions due to life-threatening arrhythmias or cardiogenic shock. Therefore, clinicians must always consider the potential causes of MINOCA, especially during the initial stages, and devise treatment strategies tailored to the underlying mechanism. Early identification of this patient group is thus crucial. Traditional coronary artery disease (CAD) risk factors such as dyslipidemia, hypertension, diabetes, smoking, and a family history of myocardial infarction were observed less frequently in MINOCA patients compared to MI-CAD patients [33]. Consistently, our findings demonstrated that smoking prevalence was significantly higher in the MI-CAD group compared to the MINOCA group. Moreover, while smoking is also expected to contribute to the MINOCA clinical profile, its role appears to be more prominent in the pathogenesis of MI-CAD. By causing endothelial dysfunction, promoting atherosclerotic plaque formation and destabilization, platelet activation, and heightened thrombotic potential, smoking is a well-established contributor to myocardial infarction and emerged as a strong independent predictor of a MI-CAD diagnosis. Additionally, although not statistically significant, MINOCA patients tended to be younger and included a higher ratio of females compared to MI-CAD patients. This epidemiological remark suggests a higher prevalence of MINOCA in these populations. Regarding physical examination findings, hair greying score (grade 3 and above) and hair loss grade (grade 4 and above) were significantly higher in MI-CAD patients compared to MINOCA patients. Previous studies have shown an association between increased hair greying and hair loss with atherosclerotic coronary artery disease [34]. However, when regarded inversely, premature hair greying and hair loss might be more strongly associated with MINOCA. Further research is needed to establish this hypothesis.

Echocardiographic results revealed that the ejection fraction in the MI-CAD group was significantly lower compared to the MINOCA group. This observation is attributed to the more heterogeneous nature of the MINOCA disease group and the relatively less obstructed coronary flow in the macro-vessel bed, leading to less apparent EF reduction. Consequently, this condition appears to provide the MINOCA group with a survival advantage compared to the MI-CAD group. While EF is an independent parameter predicting mortality in CAD and heart failure, myocardial damage in MINOCA patients may be more severe than anticipated, and long-term follow-ups may yield more accurate insights. Troponin levels may not provide sufficient information to confirm this prediction, and oxidative stress parameters as markers of cellular damage could be more informative. On the other hand, epicardial adipose tissue (EAT) is one of the most significant risk factors for atherosclerosis and cardiovascular events. EAT thickness was shown to be correlated positively with the severity of CAD, the instability of atherosclerotic plaques, aortic sclerosis, left ventricular hypertrophy, ventricular arrhythmias, myocardial infarction, and the prevalence of systolic heart failure [35]. In our study, the EAT thickness in the MI-CAD group was also found to be significantly higher. Thereby, we think the contribution of EAT to atherosclerosis is predominant compared with its contribution to coronary spasm, microvascular dysfunction, plaque disruption, and spontaneous coronary artery dissection. Additionally, the number of patients with aortic valve sclerosis was significantly higher in the MI-CAD group compared to the MINOCA group. Furthermore, TAPSE, lateral a’, and lateral e’ values, as well as right atrial and right ventricular diameters, were significantly higher in the MINOCA group compared to the MI-CAD group. These findings suggest that, in MI-CAD patients, diastolic function and right ventricular performance are more adversely affected along with left ventricular EF.

As study limitations, although the sample size in our study was appropriate for statistical analysis, the study population was relatively small and conducted in a single center. Additionally, routine use of advanced imaging modalities such as intravascular ultrasound (IVUS), optical coherence tomography (OCT), or cardiac MRI was not available for MINOCA patients, limiting the ability to obtain more detailed and reliable etiological information. Analyzing electrocardiographic data could have provided further insights. Furthermore, comparing both inflammatory and oxidative stress parameters with individuals proven to have no coronary artery disease might have yielded more enlightening results.

## 5. Conclusions

Our study demonstrated that while inflammatory parameters did not significantly differ between MI-CAD and MINOCA patients, oxidative stress parameters were significantly elevated in the MI-CAD group. Oxidative biomarkers may have utility in the risk stratification and diagnosis of myocardial infarction and for identifying patients who require closer monitoring. This study also highlights the potential value of evaluating concentrations of dityrosine, kynurenine, malondialdehyde, and lipid hydroperoxides in differentiating myocardial infarction patients with and without obstructive coronary arteries. Additionally, these markers could be employed as therapeutic targets.

## Figures and Tables

**Table 1:** Demographic and physical examination findings of patients.

Variable	All Patients (n = 88)	MINOCA (n = 44)	MI-CAD (n = 44)	*p*-Value
Demographic Data				
Age (years)	60.4 ± 11.7	56.2 ± 12.5	64.7 ± 9.3	0.001
Male sex, n (%)	60 (68.2%)	26 (59.1%)	34 (77.3%)	0.054
BMI (kg/m^2^)	29.5 ± 4.5	30.5 ± 4.1	28.6 ± 4.7	0.052
Waist circumference (cm)	103.5 ± 13.1	104.8 ± 11.6	102.1 ± 14.4	0.322
Hypertension, n (%)	48 (54.2%)	25 (56.8%)	23 (52.3%)	0.415
Diabetes mellitus, n (%)	25 (28.4%)	10 (22.7%)	15 (34.1%)	0.172
Smoking, n (%)	37 (42%)	13 (29.5%)	24 (54.5%)	0.015
Dyslipidemia, n (%)	15 (17%)	7 (15.9%)	8 (18.2%)	0.500
History of CAD, n (%)	21 (23.9%)	5 (11.4%)	16 (36.4%)	0.006
Physical Examination Findings				
Xanthelasma, n (%)	2 (2.3%)	0 (0%)	2 (4.5%)	0.247
Earlobe crease, n (%)	60 (68.2%)	31 (70.5%)	29 (65.9%)	0.410
Anterior tragal crease, n (%)	61 (69%)	29 (65%)	32 (72%)	0.322

Abbreviations: MINOCA: Myocardial Infarction with Non-Obstructive Coronary Arteries, BMI: body mass index, MI-CAD: Myocardial Infarction with Coronary Artery Disease.

**Table 2:** Echocardiographic variables of patients.

Variables	All Patients (n = 88)	MINOCA (n = 44)	MI-CAD (n = 44)	*p*-Value
Ejection fraction (%)	60 (50–60)	60 (60–60)	53 (43–60)	<0.001
End-diastolic diameter (mm)	49 (47–51)	49 (46–52)	49 (47–51)	0.712
End-systolic diameter (mm)	32 (30–36)	32 (29–35)	33 (31–36)	0.285
IVS thickness (mm)	1.1 (1–1.3)	1.1 (1–1.2)	1.2 (1.1–1.3)	0.040
LVPW thickness (mm)	1 (1–1.2)	1.1 (1–1.1)	1.1 (1–1.2)	0.064
Epicardial adipose tissue (mm)	0.4 (0.3–0.5)	0.3 (0.3–0.4)	0.5 (0.4–0.6)	<0.001
Left atrial diameter (mm)	37 (34–40)	37 (34–39)	36 (34–40)	0.821
Left atrium max volume (mL)	41 (29–56)	44 (33–56)	35 (27–49)	0.054
Left atrium min volume (mL)	17 (13–25)	18 (13–24)	16 (12–28)	0.748
LAVI (mL/m^2^)	1.35 (1.05–1.76)	1.44 (1.07–1.86)	1.15 (0.98–1.73)	0.154
Mitral E wave	0.68 (0.57–0.84)	0.71 (0.57–0.83)	0.67 (0.57–0.85)	0.507
Mitral A wave	0.78 (0.66–0.93)	0.74 (0.59–0.92)	0.79 (0.72–0.97)	0.077
Deceleration time (ms)	175 (145–218)	175 (146–219)	173 (140–218)	0.646
Lateral a’	0.1 (0.09–0.12)	0.11 (0.09–0.13)	0.1 (0.08–0.12)	0.024
Lateral e’	0.09 (0.07–0.12)	0.1 (0.08–0.13)	0.08 (0.06–0.1)	0.012
MPI	0.59 (0.55–0.66)	0.59 (0.55–0.64)	0.59 (0.54–0.72)	0.473
Ascending aorta diameter (mm)	37 (33–39)	36 (33–39)	37 (33–39)	0.749
Aortic valve sclerosis n (%)	20 (22%)	6 (13.6%)	14 (31%)	0.037
MAC n (%)	5 (5.7%)	3 (7%)	2 (4.5%)	0.500
Presystolic wave n (%)	61 (69%)	28 (63%)	33 (75%)	0.178
TAPSE	20 (18–21)	20 (19–21.7)	19 (17–21)	0.043
Right atrial diameter (mm)	33 (31–35.7)	33.5 (32–37)	33 (30–34)	0.026
Right ventricular diameter (mm)	29 (26.2–31.7)	30 (27.5–33)	28 (24–30)	0.004
Right atrial max volume (mL)	30 (24–38)	34.5 (26–44)	27 (20–33)	0.001

Abbreviations: IVS, interventricular septal thickness; LVPW, left ventricular posterior wall thickness; EAT, epicardial adipose tissue; LA, left atrium; LAVI, left atrial volume index; DT, deceleration time; MPI, myocardial performance index; AAO, ascending aorta; AVS, aortic valve sclerosis; MAC, mitral annular calcification; PPW, presystolic wave; TAPSE, tricuspid annular plane systolic excursion, MI-CAD: Myocardial Infarction with Coronary Artery Disease.

**Table 3:** Laboratory findings of patients.

Variables	All Patients (n = 88)	MINOCA (n = 44)	MI-CAD (n = 44)	*p*-Value
WBC (10^3^/uL)	8.81 ± 2.61	8.47 ± 2.51	9.16 ± 2.69	0.222
Hemoglobin (g/L)	13.7 (12.7–14.7)	14 (13.3–15)	13.5 (11.9–14.6)	0.075
Neutrophil (10^3^/uL)	5.2 (3.7–7.2)	4.6 (3.4–7.1)	5.9 (4.1–8.2)	0.094
Lymphocyte (10^3^/uL)	2.3 (1.7–2.8)	2.5 (1.6–3)	2.2 (1.9–2.6)	0.319
Platelets (/mm^3^)	232.5 ± 60.6	235.2 ± 60.9	229.7 ± 60.9	0.671
Triglycerides (mg/dL)	129 (95–188)	140 (104–206)	116 (87–184)	0.127
HDL (mg/dL)	39.5 (34–48)	40.1 (34.1–51.8)	40.4 (34–48)	0.478
LDL (mg/dL)	133.5 ± 47.6	135.1 ± 47.1	131.8 ± 48.7	0.746
Sodium (mEq/L)	137 (136–139)	137.5 (136–139)	137 (136–139)	0.525
Potassium (mEq/L)	4.22 ± 0.46	4.15 ± 0.45	4.3 ± 0.47	0.133
BUN (mg/dL)	35.5 (29–44)	35 (30–42.5)	37 (28–47)	0.622
AST (IU/L)	26.5 (19–46)	27 (20–38)	24 (19–55)	0.841
ALT (IU/L)	20.5 (14–31)	20 (14.5–37)	22 (14–31)	0.757
Total Protein (mg/dL)	72 (67–74)	73 (70–76)	69 (66–73)	<0.001
Albumin (mg/dL)	42 (40–45)	42.5 (40.2–46)	42 (39–44)	0.105
CRP (mg/dL)	5.8 (2.8–11.2)	5.7 (3.2–6.9)	6.9 (2.2–13.1)	0.792

Abbreviations: WBC: White Blood Cell, HDL: High-Density Lipoprotein, LDL: Low-Density Lipoprotein, BUN: Blood Urea Nitrogen, AST: Aspartate Aminotransferase, ALT: Alanine Aminotransferase, CRP: C-Reactive Protein, MI-CAD: Myocardial Infarction with Coronary Artery Disease.

**Table 4:** Inflammatory and oxidative stress status of patients.

Variable	All Patients (n = 88)	MINOCA (n = 44)	MI-CAD (n = 44)	*p*-Value
Immunity-Inflammation Parameters				
WBC (10^3^/uL)	8.81 ± 2.61	8.47 ± 2.51	9.16 ± 2.69	0.222
CRP (mg/dL)	5.8 (2.8–11.2)	5.7 (3.2–6.9)	6.9 (2.2–13.1)	0.792
NLR	2.1 (1.6–3.5)	1.9 (1.4–3.3)	2.7 (1.8–3.9)	0.054
SII	548 (343–905)	421 (286–819)	574 (402–934)	0.100
TLR	101 (77–136)	98 (75–137)	105 (85–133)	0.515
CAR	1.4 (0.6–2.6)	1.3 (0.7–1.6)	1.5 (0.5–3)	0.690
ACR	5.1 ± 1.4	5.3 ± 1.5	5 ± 1.3	0.421
PNI	42 (40–45)	42.5 (40.5–46)	42 (39–44)	0.090
Oxidative Stress Parameters				
Dityrosine (FU/mL)	5307 (4406–6161)	4488 (3641–5238)	5708 (5311–6417)	<0.001
Lipid hydroperoxide (nmol/mL)	3.6 (3.3–3.9)	3.4 (3.1–3.9)	3.6 (3.4–3.9)	0.023
Malondialdehyde (nmol/mL)	14.3 (12.4–17.8)	13.1 (12–14.9)	17.4 (13.7–19.1)	<0.001
Kynurenine (FU/mL)	3625 (3005–4133)	3206 (2853–3765)	3954 (3317–4181)	0.001

Abbreviations: ACR: Albumin/Creatinine Ratio, CAR: CRP/Albumin Ratio, PNI: Prognostic Nutritional Index, SII: Systemic Immune-Inflammatory Index, NLR: Neutrophil-to-Lymphocyte Ratio, TLR: Thrombocyte-to-Lymphocyte Ratio, MI-CAD: Myocardial Infarction with Coronary Artery Disease.

**Table 5:** Independent predictors of Myocardial Infarction with Coronary Artery Disease.

Variable	Univariate Analysis (OR, 95% CI)	*p*-Value	Multivariable Analysis (OR, 95% CI)	*p*-Value
Age	1.073 (1.026–1.122)	0.002	1.195 (1.042–1.371)	0.011
Gender (male)	2.354 (0.932–5.945)	0.070	-	-
Body mass index	0.917 (0.883–1.010)	0.079	-	-
Smoking	2.862 (1.189–6.888)	0.019	76.919 (4.665–1263.774)	0.002
Hair Greying Score (moderate–severe)	5.172 (1.555–17.209)	0.007	-	-
Alopecia (moderate–severe)	5.286 (1.943–14.381)	0.001	-	-
Ejection fraction	0.896 (0.840–0.956)	0.001	-	-
Endothelial dysfunction	0.197 (0.078–0.501)	0.001	0.008 (0.001–0.246)	0.006
Epicardial Fat Thickness	450 (0.133–1450)	0.140	-	-
Right Atrium Maximum Volume	0.931 (0.888–0.975)	0.003	0.740 (0.607–0.902)	0.003
Dityrosine	1.001 (1.001–1.002)	0.001	1.001 (1.000–1.002)	0.011
Lipid hydroperoxide	2.420 (1.088–5.383)	0.030	-	-
Malondialdehyde	1.351 (1.155–1.579)	0.001	2.096 (1.286–3.416)	0.003
Kynurenine	1.001 (1.000–1.002)	0.004	-	-

Abbreviations: CI: Confidence Interval, OR: Odds Ratio, MI-CAD: Myocardial Infarction with Coronary Artery Disease.

**Table 6:** Independent predictors of MINOCA.

Variables	Univariate Analysis (OR, 95% CI)	*p*-Value	Multivariable Analysis (OR, 95% CI)	*p*-Value
Age	0.930 (0.889–0.973)	0.002	-	-
Gender (male)	0.425 (0.168–1.073)	0.070	-	-
Body mass index	1.098 (0.996–1.210)	0.060	-	-
Smoking	0.322 (0.133–0.777)	0.012	0.009 (0.001–0.276)	0.007
Hair Greying Score (moderate–severe)	0.193 (0.058–0.643)	0.007	-	-
Alopecia (moderate–severe)	0.189 (0.070–0.515)	0.001	-	-
Endothelial dysfunction	5.610 (2.189–14.380)	<0.001	116.079 (4.039–3336.4)	0.006
Ejection fraction	1.133 (1.056–1.215)	0.001	1.377 (1.045–1.816)	0.023
Epicardial Fat Thickness	0.120 (0.133–1320)	0.220	-	-
Right Atrium Max Volume	1.073 (1.025–1.124)	0.003	1.365 (1.093–1.705)	0.006
Dityrosine	0.999 (0.998–0.999)	<0.001	0.999 (0.998–1.000)	0.026
Lipid hydroperoxide	0.336 (0.144–0.783)	0.011	-	-
Malondialdehyde	0.751 (0.644–0.875)	<0.001	0.607 (0.382–0.963)	0.034
Kynurenine	0.999 (0.998–1.000)	0.001	-	-

Abbreviations: CI: Confidence Interval, OR: Odds Ratio.

## Data Availability

The data presented in this study are available on request from the corresponding author. The data are not publicly available due to privacy or ethical restrictions arising from patient confidentiality.
